# Behavioural and demographic correlates of undiagnosed HIV infection in a MSM sample recruited in 13 European cities

**DOI:** 10.1186/s12879-018-3249-8

**Published:** 2018-08-06

**Authors:** Ulrich Marcus, Christiana Nöstlinger, Magdalena Rosińska, Nigel Sherriff, Lorenzo Gios, Sonia F. Dias, Ana F. Gama, Igor Toskin, Ivailo Alexiev, Emilia Naseva, Susanne Barbara Schink, Massimo Mirandola, the Sialon II Network, Massimo Mirandola, Lorenzo Gios, Stefano Benvenuti, Ruth Joanna Davis, Silvana Menichelli, Michele Breveglieri, Wim Vanden Berghe, Peter de Groot, Christiana Nöstlinger, Veronica van Wijk, Katrien Fransen, Tine Vermoesen, Michiel Vanackere, Fourat Benchikha, Sandra van den Eynde, Boris Cruyssaert, Mark Sergeant, Karel Blondeel, Pieter Damen, François Massoz, Erwin Carlier, Michael François, Stephen Karon, Safia Soltani, Thierry Martin, Alan De Bruyne, Francoise Bocken, Myriam Dieleman, Ivailo Alexiev, Reneta Dimitrova, Anna Gancheva, Dobromira Bogeva, Maria Nikolova, Mariya Muhtarova, Todor Kantarjiev, Viara Georgieva, Emilia Naseva, Petar Tsintsarski, Hristo Taskov, Tonka Varleva, Elena Birindjieva, Aneliya Angelova, Manol Antonov, Ulrich Marcus, Susanne Barbara Schink, Sandra Dudareva-Vizule, Matthias an der Heiden, Sami Marzougui, Viviane Bremer, Andrea Kühne, Kerstin Schönerstedt-Zastrau, Ruth Zimmermann, Andreas Wille, Kai Eckstein, Norman Buch, Philipp Moskophidis, Marc Grenz, Danilo Schmogro, Giuseppe Cornaglia, Antonella Zorzi, Elisabetta Tonolli, Giuliana Lo Cascio, Teresa Todeschini, Manuela Recchia, Lorella Pattini, Maria Rocca, Alessandra Bighignoli, Anita Galardi, Loredana Martini, Francesco Cobello, Chiara Bovo, Giulia Bisoffi, Oscar Bortolami, Laura Crestani, Fabiano Comperini, Ercole Concia, Emanuela Lattuada, Massimiliano Lanzafame, Paola Del Bravo, Maddalena Cordioli, Fabio Rigo, Emanuele Guardalben, Ivan Marchesoni, Barbara Suligoi, Vincenza Regine, Lucia Pugliese, Saulius Caplinskas, Irma Caplinskiene, Rima Krupenkaite, Gediminas Sargelis, Arturas Rudomanskis, Sónia Dias, Ana Gama, Oriana Brás, João Piedade, Ricardo Fuertes, Nuno Pinto, João Brito, Júlio Esteves, Jesus Rojas, Fernando Ferreira, Miguel Rocha, Hugo Machado, Maria José Campos, Luís Mendão, Magdalena Rosińska, Bożena Kucharczyk, Marta Niedźwiedzka-Stadnik, Łukasz Henszel, Andrzej Zieliński, Michał Czerwiński, Michał Pawlęga, Ewelina Burdon, Małgorzata Gajdemska, Agnieszka Guściora, Nikodem Klasik, Katarzyna Rżanek, Michał Sawicki, Michał Tęcza, Ewelina Burdon, Małgorzata Gajdemska, Agnieszka Guściora, Nikodem Klasik, Katarzyna Rżanek, Mateusz Dębski, Anna Maciejewska, Izabela Pazdan, Izabela Pazdan, Alexandru Rafila, Daniela Pitigoi, Adrian Abagiu, Carolina Marin, Ioana Panzariu, Alexandru Miroiu, Madalina Popa, Monica Likker, Maria Georgescu, Galina Musat, Dan Cojocaru, Mihai Lixandru, Raluca Teodorescu, Danica Staneková, Monika Hábeková, Tatiana Drobková, Zuzana Chabadová, Soňa Wimmerova, Maria Mojzesová, Filip Kunč, Michal Skurák, Peter Bodnar, Katarína Horniaková, Mária Krahulcová, Jarmila Präsensová, Martin Smoleň, Peter Záhradník, Pavol Tibaj, Irena Klavs, Tanja Kustec, Claudia Adamič, Mario Poljak, Robert Krošelj, Jana Mlakar, Miran Šolinc, Cinta Folch, Laia Ferrer, Alexandra Montoliu, Jordi Casabona, Anna Esteve, Montserrat Galdon, Victoria Gonzalez, Rafael Muñoz, Maria Axelsson, Torsten Berglund, Sharon Kuhlmann-Berenzon, Achilleas Tsoumanis, Inga Velicko, Christer Janson, Bartek Lindh, Kajsa Aperia, Buddha Babulanam, Hans Carlberg, Malte Davidsson, Nedo Entenza Gutierrez, Viktor Hildingsson, Henrik Klasson, Moises Peña Ramos, Cristian Quintero Rojas, Sven-Olof Sandberg, Andreas Samuelson, Eric Sjöberg, Tommy Sjölund, Simon Svensson, Iván Valencia, Filip Garcia, Olov Lindblad, Jon Voss, Ronnie Ask, Anders Blaxhult, Maarit Maliniemi, Monica Ideström, Nils Blom, Nigel Sherriff, Christina Panton, Glynis Flood, Katrien Fransen, Tine Vermoesen, Ross Boseley, Marc Tweed, Jonathon Roberts, Cinthia Menel Lemos, Paolo Guglielmetti, Wolfgang Philipp, Matthias Schuppe, Andrew Amato, Irina Dinca, Karin Haar, Anastasia Pharris, Teymur Noori, Igor Toskin, Armando Seuc, Natalie Maurer, Lev Zohrabyan, Alexandrina Iovita, Maddalena Campioni, Patrick Noack, Rosanna Peeling, Lisa Johnston

**Affiliations:** 1 Department of Infectious Diseases Epidemiology, Robert Koch-Institute, Berlin, Germany; 2 Department of Public Health, Institute of Tropical Medicine, Antwerp, Belgium; 3 Department of Epidemiology, National Institute of Public Health, Warsaw, Poland; 4 Health Sciences, University of Brighton, Brighton, UK; 5 Infectious Diseases Section, Department of Diagnostics and Public Health, University of Verona, Verona, Italy; 6 Escola Nacional de Saúde Pública Universidade, Centro de Investigação em Saúde Pública, Universidade Nova de Lisboa, Lisbon, Portugal; 7 Instituto de Higiene e Medicina Tropical, Global Health and Tropical Medicine, Universidade Nova de Lisboa, Lisbon, Portugal; 8 Department of Reproductive Health and Research, World Health Organization, Geneva, Switzerland; 9 National Centre of Infectious and Parasitic Diseases, National Reference Laboratory of HIV, Sofia, Bulgaria; 10 Ministry of Health, Program “Prevention and control of HIV/AIDS”, Sofia, Bulgaria

**Keywords:** HIV, Men having sex with men, Europe, Biobehavioural survey, Undiagnosed HIV infection

## Abstract

**Background:**

Reducing the number of people with undiagnosed HIV infection is a major goal of HIV control and prevention efforts in Europe and elsewhere. We analysed data from a large multi-city European bio-behavioural survey conducted among Men who have Sex with Men (MSM) for previously undiagnosed HIV infections, and aimed to characterise undiagnosed MSM who test less frequently than recommended.

**Methods:**

Data on sexual behaviours and social characteristics of MSM with undiagnosed HIV infection from Sialon II, a bio-behavioural cross-sectional survey conducted in 13 European cities in 2013/2014, were compared with HIV-negative MSM. Based on reported HIV-testing patterns, we distinguished two subgroups: MSM with a negative HIV test result within 12 months prior to the study, i.e. undiagnosed incident infection, and HIV positive MSM with unknown onset of infection. Bivariate and multivariate associations of explanatory variables were analysed. Distinct multivariate multi-level random-intercept models were estimated for the entire group and both subgroups.

**Results:**

Among 497 participants with HIV-reactive specimens, 234 (47.1%) were classified as previously diagnosed, 106 (21.3%) as incident, and 58 (11.7%) as unknown onset based on self-reported status and testing history. MSM with incident HIV infection were twice as likely (odds ratio (OR) = 2.22, 95% confidence interval (95%CI): 1.17–4.21) to have used recreational substances during their last anal sex encounter and four times more likely (OR = 3.94, 95%CI: 2.14–7.27) not to discuss their HIV status with the last anal sex partner(s). MSM with unknown onset of HIV infection were 3.6 times more likely (OR = 3.61, 95%CI: 1.74–7.50) to report testing for a sexually transmitted infection (STI) during the last 12 months.

**Conclusions:**

Approximately one third of the study participants who are living with HIV were unaware of their infection. Almost two-third (65%) of those with undiagnosed HIV appeared to have acquired the infection recently, emphasizing a need for more frequent testing. Men with the identified behavioural characteristics could be considered as primary target group for HIV Pre-Exposure Prophylaxis (PrEP) to avoid HIV infection. The increased odds of those with unknown onset of HIV infection to have had an STI test in the past year strongly suggests a lost opportunity to offer HIV testing.

**Electronic supplementary material:**

The online version of this article (10.1186/s12879-018-3249-8) contains supplementary material, which is available to authorized users.

## Background

In recent years a range of efforts and new initiatives have been implemented across Europe to increase Human Immunodeficiency Virus (HIV) testing among key populations and to reduce the number of undiagnosed HIV infections and late diagnoses [1]. Despite some progress in terms of increased testing uptake, a recent report from the European Centre for Disease Prevention and Control (ECDC) estimates that the proportion of undiagnosed HIV among Men having Sex with Men (MSM) in six European countries is 17% [2]. This falls short of the internationally agreed goal of diagnosing at least 90% of the people who are infected [3]. Furthermore, approximately one third of HIV diagnoses among MSM in the European Union (EU) are late (CD4 < 350 cells/μl at diagnosis). Reducing late HIV diagnoses would result in substantial individual (i.e. reduced morbidity and mortality) and public health benefits (reduced transmission and reduction of health care costs) [4, 5]. In addition to adverse health outcomes, late diagnoses and late initiation of antiretroviral therapy are associated with increased risks for transmitting HIV unknowingly to sexual partners.

In countries with unrestricted access to antiretroviral treatment undiagnosed HIV infections are thought to be the main sources of new HIV infections. Early diagnosis of HIV and successful treatment are thus important for the successful management of the disease in individual patients as well as major tools supporting the implementation of the WHO strategy for anti-retroviral treatment as prevention [3, 6, 7].

The level of undiagnosed infections is driven by HIV incidence on the one hand and by the testing rate on the other. Factors increasing HIV incidence are likely to contribute to the increased level of undiagnosed infections, even among frequent testers. Additionally, barriers to testing and low testing uptake may cause accumulation of infections, including late stage infections [8, 9].

Little is known about the characteristics and sexual behaviours of people with undiagnosed infections. Some information can be retrieved from HIV testing sites from people newly diagnosed with HIV [10–12], although the demographic data collected and analysed are quite limited from such clinical sites [13, 14]. People presenting for testing self-select and therefore such samples may be biased. Another way to collect such information is from longitudinal cohort studies including HIV-uninfected people at risk for HIV. However, such studies are time consuming, costly, and rarely conducted in Europe. In addition, participants may not be representative of the population at risk in real world settings. Bio-behavioural studies, such as the multi-city Sialon II study, may therefore be better suited to systematically collect information on people who are unknowingly infected with HIV [15, 16] with more scientific rigour and fewer biases.

In general, the risk of HIV infection among MSM is associated with sexual risk practices such as the lack of condom use, number of partners with whom condomless anal intercourse is practised, drug use associated with sex, and attending gay sex venues where risky sexual behaviours are part of the sub-culture [17]. However, such risk practices are driven by a complex set of intertwined factors, ranging from the personal level factors (e.g. age, personal skills and self-efficacy, mental health) to interpersonal factors (partner dynamics, communication and negotiation on sex practices), to community and service provision-related factors (social and sexual norms, perceived homonegativity in communities; access to testing and other medical services) and structural factors (policies and legislation). The intersection of these factors is further shaped by high HIV prevalence in subgroups of MSM [18].

Barriers to HIV testing have also been extensively described in the literature: testing for HIV is more likely for individuals who perceive themselves at risk for HIV and who anticipate personal benefits from testing, while fears of consequences of receiving an HIV diagnosis hinders HIV testing. The latter has been shown to be associated with fear of discrimination and personal rejection [19, 20]. Research has also shown that multiple social-cognitive factors (e.g. knowledge, attitudes, perceived behavioural control) play a role in explaining testing for HIV and sexually transmitted infections (STI) among MSM [20]. In addition to patient-level factors, a review demonstrated the influence of health-systems and structural factors on uptake of HIV testing [8].

The present study used data from a large multi-city European bio-behavioural survey conducted among MSM within the framework of the European Public Health Project Sialon II. The analysis has two objectives – 1) to identify factors correlating with early undiagnosed infections among testers, which are most likely driven by HIV incidence among repeat testers; and 2) to characterise groups that are not adhering to testing recommendations in order to properly inform appropriate testing campaigns targeted towards them.

## Methods

### Study design and procedures

Sialon II was a multi-site bio-behavioural cross-sectional survey carried out in 13 European cities*.* The cities were: Brussels (Belgium), Sofia (Bulgaria), Hamburg (Germany), Verona (Italy), Vilnius (Lithuania), Warsaw (Poland), Lisbon (Portugal), Bucharest (Romania), Bratislava (Slovakia), Ljubljana (Slovenia), Barcelona (Spain), Stockholm (Sweden), and Brighton (UK). In 2013/2014, MSM were recruited to participate in the survey using time-location-sampling (TLS) in community-based settings in nine European cities, and using respondent-driven-sampling (RDS) in social networks of MSM in four European cities (Bucharest, Bratislava, Verona, Vilnius). In TLS cities participants were recruited during 2013, in RDS cities recruitment started in 2013 and finished in 2014. Recruitment methods, study procedures, questions asked as well as sample collection and testing have been described in detail elsewhere [21]. The study protocol was approved by ethical review committees in all participating countries and by the WHO Research Project Review Panel (WHO-RP2) and the WHO Research Ethics Review Committee (WHO-ERC) before the data collection phase.

The bio-behavioural survey data generated from the Sialon II project provided the opportunity to combine data on testing history and self-reported HIV test result, and to link them with the laboratory determined HIV status to identify men with an undiagnosed HIV infection at the time of the survey implementation. In this analysis, sexual behaviours and social characteristics of these men with undiagnosed HIV infection are assessed and compared with their uninfected peers.

### Measures

Participants filled in a short questionnaire and provided either an oral fluid (in TLS cities) or blood (in RDS cities) specimen for HIV antibody testing. Based on self-reported HIV status and the HIV testing result of the collected specimen, participants were classified as HIV-uninfected (nHIV), previously diagnosed with HIV infection (pHIV), and HIV-infected but as yet undiagnosed (uHIV). As far as the time of HIV diagnosis is concerned, three different patterns can be distinguished: 1) early diagnosis, when testing is predominantly triggered by symptoms of acute HIV infection and/or awareness of transmission risk; 2) intermediate diagnosis, when testing is triggered by health concerns not immediately related to acute HIV disease or transmission risk awareness; 3) late diagnosis, often triggered by symptoms or health complaints associated with compromised immune status. These three patterns may be associated with different demographic and behavioural characteristics (see below). Since data on HIV testing intentions were not collected, we used HIV testing history to distinguish between a group with likely high testing frequency and incident HIV infection (uHIVinc - negative test result reported within 12 months before the study specimen was collected) and MSM who were tested a longer time ago or never tested for HIV infection of unknown onset (uHIVunk). The uHIVinc subgroup may represent pattern 1 and partially pattern 2 testers, while the uHIVunk subgroup may represent the complementary part of pattern 2 and pattern 3 testers.

The following questionnaire items were used to determine if the case had been previously diagnosed: a question whether and if yes, when the last HIV antibody test was performed and a question on the result of the last HIV antibody test. If these questions were not answered or information was inconsistent HIV status knowledge was classified as undetermined and respondents were excluded from the analysis. In addition, participants recruited in Sofia had to be excluded from the two test recency subgroup analyses because the answers were invalid due to an incorrect translation of the respective question.

### Lab testing of biological samples

In line with the TLS protocols, oral fluid (OF) specimens were collected and tested for HIV antibodies using Genscreen HIV 1/2 version 2, BIO-RAD. A total IgG antibodies ELISA test Human IgG ELISA Kit 1 × 96, Quantitative / Immunology Consultants Laboratory was used for OF specimen testing suitability and quality control. All HIV-reactive specimens were re-tested with Vironostika HIV Ag/Ab, bioMérieux. Specimens tested positive with the first HIV ELISA test, but negative with the second were classified as negative.

MSM who participated to the survey in cities where RDS was used as recruitment method received pre/post-test counselling during the enrolment and follow up process. Blood samples were collected and serum extracted in line with the local standard procedures. Serum samples were tested with an HIV 4th generation ELISA/CLIA screening test. A Western blot test was used to confirm positive cases. In case of a confirmed HIV positive result, a referral procedure was put in place in line with the local standard procedures to ensure linkage to care and proper case management.

### Secondary variables

Based on published literature on factors associated with HIV acquisition risk, undiagnosed HIV infection or infrequent HIV testing and late diagnosis among MSM (as mentioned in the introduction), associations of uHIV, uHIVinc and uHIVunk status were analysed with:

- Demographic variables such as age (calculated using the self-reported year of birth), education level (secondary school or lower, high school or post-secondary or university/ higher), migration status (native: born & living in the study country; emigrant: born in the study country & living abroad; immigrant: born abroad & living in the study country; visitor: born & living abroad);
- Behavioural variables such as number of sexual partners and number of partners with whom condomless anal intercourse (AI) had been practiced in the previous 6 months, frequency of visiting gay sex venues in the last 3 months, type and number of drugs used during last AI (categorised as alcohol; cannabis; sexual performance enhancing substances: erectile dysfunction medication and inhaled amyl nitrite; party drugs: cocaine, ecstasy, amphetamines; chemsex drugs: GHB, ketamine, mephedrone, crystal meth);
- Type of partners for last AI (steady, non-steady, more than one), self-reported HIV serostatus disclosure to the last AI partner, sexual role during last AI (top, bottom, versatile), condom use during last AI;
- HIV and STI testing in the previous 12 months, and “outness” about sexual orientation towards relatives, friends, and co-workers.

The self-administered questionnaire filled-in by the study participants is available as Additional file 1.

### Statistical analysis

We conducted analyses of bivariate and multivariate associations of explanatory variables with uHIV, and the two subgroups uHIVinc and uHIVunk, using nHIV for comparison. For the two subgroups, the comparison group was also determined by their last reported HIV test date, i.e. the comparison group for uHIVinc was tested negative within the previous 12 months, and the comparison group for uHIVunk was never tested or tested more than 12 months ago.

A multivariate multi-level logistic random-intercept model (random effect of study site) was estimated to account for the hierarchical structure of the data [22]. The multi-level analysis was conducted to identify factors associated with each subgroup separately and with the combined group. Predictors associated with the outcome variable with a probability < 0.05 were considered significant.

Stata Version 14.2 was used (College Station, TX: StataCorp LP).

The dataset used for the analysis presented in this manuscript is available as Additional file 2. The Stata syntax of the analysis is available as Additional file 3.

## Results

### Study sample

A detailed description of the study sample has been published in the study report [23]. At most study sites, approximately 400 men had been recruited as requested by the study protocol, with exception of Bucharest, where only 183 participants were enrolled. There were significant age differences between study sites. The proportion of study participants tested for HIV in the last 12 months before completing the study questionnaire among those not known to have been diagnosed with HIV ranged between 35.5% in Bratislava and 66.2% in Barcelona (see Table 1).

**Table 1:** Sialon II study participants by study site, testing history and measured HIV status after exclusion of participants already known to have HIV and with indeterminate HIV status knowledge

city	HIV negative (n)	percentage tested for HIV in the last 12 months (%)	undiagnosed HIV infection (n)	percentage with undiagnosed HIV (%)	undiagnosed HIV, negative HIV pre-test within recent 12 months (n)	undiagnosed HIV infection, no pre-test or test longer ago than 12 months (n)	percentage of undiagnosed HIV that may not be recent (%)	Total (N)
Barcelona	334	66.2%	21	5.9%	16	5	23.8%	355
Bratislava	376	35.5%	15	3.8%	6	9	60.0%	391
Brighton	331	56.7%	15	4.3%	10	5	33.3%	346
Brussels	327	63.5%	7	2.1%	5	2	28.6%	334
Bucharest	146	42.9%	15	9.3%	7	8	53.3%	161
Hamburg	336	52.1%	15	4.3%	11	4	26.7%	351
Lisbon	300	63.8%	29	8.8%	23	6	20.7%	329
Ljubljana	329	50.9%	7	2.1%	5	2	28.6%	336
Sofia^a^	344		12	3.4%				356
Stockholm	334	49.9%	3	0.9%	2	1	33.3%	337
Verona	367	41.9%	10	2.7%	2	8	80.0%	377
Vilnius	314	38.9%	5	1.6%	2	3	60.0%	319
Warsaw	346	57.9%	22	6.0%	17	5	22.7%	368
Total	4184		176	4.0%				4360
Total w/o Sofia	3840	51.9%	164		106	58	33.0%	4004

^a^data on recency of last HIV testing are missing for Sofia due to incorrect translation of the question in the Bulgarian questionnaire version

Formative research conducted in preparation of the bio-behavioural survey established that HIV testing sites, including sites providing free and anonymous HIV testing and rapid testing existed in all study cities at the time when study recruitment occurred [24]. Further qualitative assessments of gay-friendliness, accessibility and acceptability of available testing services were not conducted. HIV home tests and home collection tests were unavailable.

A valid HIV test result was available for 4716 participants. The antibody test result was non-reactive for 4219 specimens, and reactive for 497 specimens (11.8%). From the 4219 participants with non-reactive specimens 4184 (99%) were classified as nHIV, 35 were classified as indeterminate due to conflicting or missing self-reported data on HIV infection status. From the participants with reactive specimens 234 (47%) were classified as pHIV, 102 (20.5%) as uHIVinc, and 49 (9.9%) as uHIVunk based on self-reported infection status and testing history. Twelve participants from Sofia with undiagnosed HIV infection could not be classified in these two subgroups. The remaining 100 (20.1%) participants with reactive specimens had to be classified as indeterminate based on questionnaire data due to incomplete information on testing history and status knowledge (e.g. non-response to the question on previous HIV test and/or test result).

A weak positive correlation between the percentage of the participants tested for HIV by study site in the recent 12 months and the percentage of undiagnosed HIV in the study sites was observed (*r* = .275 - see Table 1).

### Undiagnosed HIV infections and associations with demographics and behaviours

**Fig. 1:**
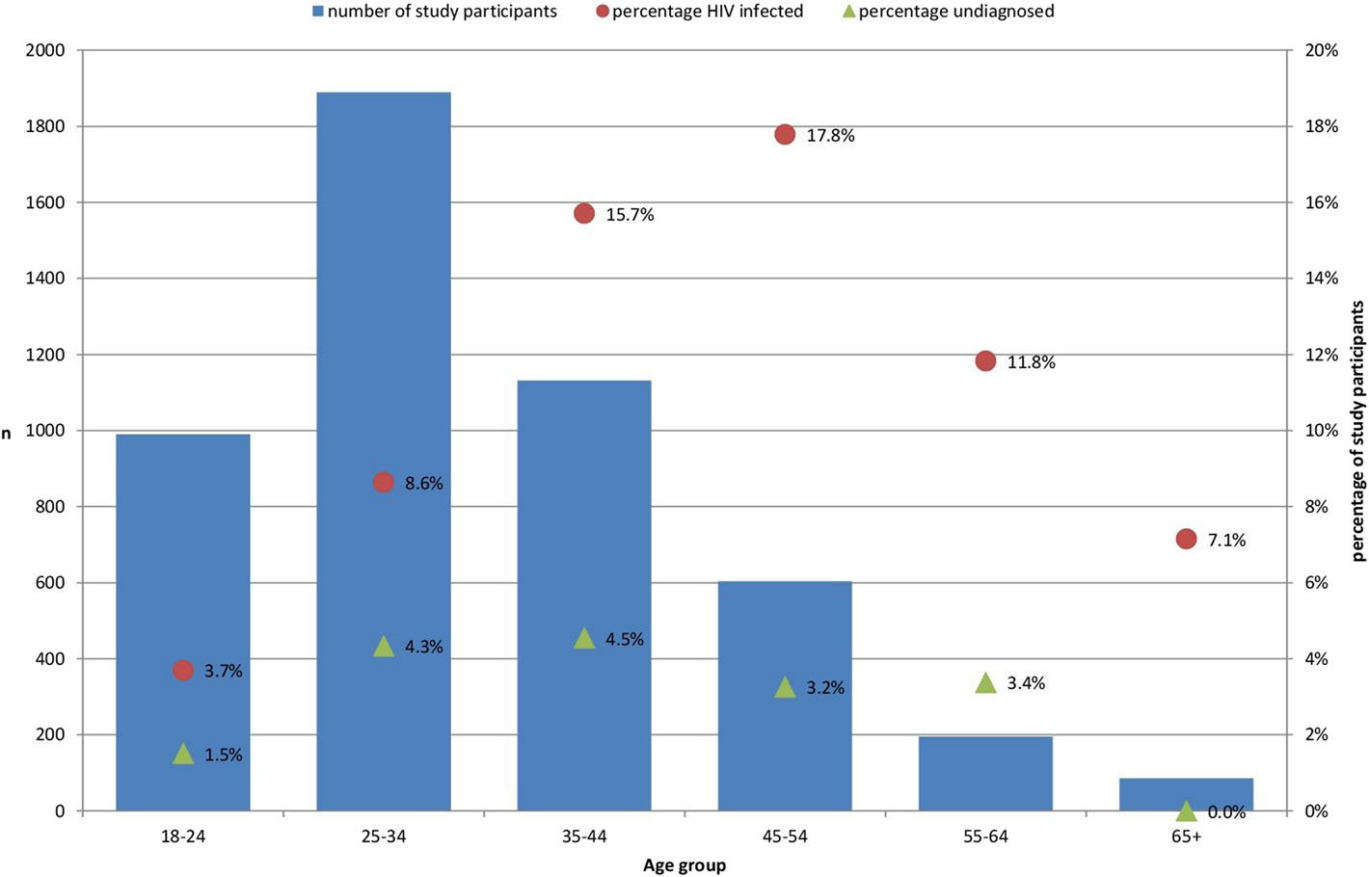
Age distribution and prevalence of HIV infection and undiagnosed HIV by age group in the Sialon II participants, Sialon II biobehavioural survey, 2013–2014

The distribution of all and of undiagnosed infections by age group is shown in Fig. 1.

The percentage of undiagnosed infections from all prevalent infections is approaching 50% in age groups younger than 35 years-old and declines to less than 30% in older age groups.

Table 1 shows the distribution of undiagnosed HIV infections by study sites. The proportion of study participants with undiagnosed HIV infection ranged from 0.9% in Stockholm to 9.3% in Bucharest. The overall proportion of undiagnosed HIV infections among men without a recent test result was almost one-third of the undiagnosed infections, ranging from 20.7% in Lisbon to 80% in Verona. The proportions of undiagnosed HIV among infrequent testers were consistently higher than 50% in the four cities Bratislava, Bucharest, Vilnius and Verona, in which RDS was used for recruitment.

Table 2 shows the reported last test dates among study participants who did not report having HIV or a last HIV test within the 12 months before they were recruited to the Sialon study.

**Table 2:** Distribution of Sialon II study participants who were not aware of having HIV by laboratory-determined HIV status and year of last HIV test - overall and by test recency group

uHIV - study sample			uHIVunk - last HIV test not within 12 months of recruitment into the study		
Year of last reported test	HIV negative	HIV positive, undiagnosed	Year of last reported test	HIV negative	HIV positive, undiagnosed
1987	1	0	1987	1	0
1988	1	0	1988	1	0
1989	2	0	1989	2	0
1990	5	0	1990	5	0
1991	1	0	1991	1	0
1992	3	0	1992	3	0
1993	2	0	1993	2	0
1994	1	0	1994	1	0
1995	4	1	1995	4	1
1996	2	1	1996	2	1
1997	5	1	1997	5	1
1998	7	0	1998	7	0
1999	6	0	1999	6	0
2000	18	0	2000	18	0
2001	10	0	2001	10	0
2002	13	1	2002	13	1
2003	15	0	2003	15	0
2004	14	0	2004	14	0
2005	39	2	2005	39	2
2006	22	2	2006	22	2
2007	39	0	2007	39	0
2008	61	0	2008	61	0
2009	80	4	2009	80	4
2010	151	13	2010	151	13
2011	267	8	2011	267	8
2012	785	47	2012	205	10
2013	1306	70	2013	30	2
2014	32	0	never tested/year missing	864	13
year missing^a^	1292	26	Total	1868	58
Total	4184	176			
			uHIVinc - last test within 12 months before recruitment into the study		
			2012	580	37
			2013	1276	68
			2014	32	0
			year missing, test within the last 12 months reported	84	1
			Total	1972	106

^a^includes 356 participants from Sofia whose last HIV test date could not be determined

Table 3 shows results of bivariate analysis of associations between potential explanatory variables and the outcomes 1) undiagnosed HIV infection, acquired within the previous 12 months - uHIVinc; 2) undiagnosed HIV infection of unknown onset - uHIVunk; 3) undiagnosed HIV infection irrespective of date of previous HIV test (1 and 2 combined - uHIV).

**Table 3:** Associations between demographic and behaviour variables and undiagnosed HIV infection in participants of the European Sialon II biobehavioural survey

		uHIVinc				uHIVunk				uHIV				N
		Odds Ratio	*p*-value	[95% Conf. Interval]		Odds Ratio	*p*-value	[95% Conf. Interval]		Odds Ratio	*p*-value	[95% Conf. Interval]		
Age group	18–24	ref.												923
	25–34	**3.34**	**0.00**	1.58	7.07	1.69	0.25	0.70	4.07	**2.65**	**0.00**	1.56	4.50	1730
	35–44	**3.05**	**0.01**	1.37	6.80	**2.82**	**0.02**	1.18	6.73	**2.90**	**0.00**	1.66	5.06	970
	45–54	2.37	0.07	0.94	5.97	2.00	0.19	0.72	5.59	**2.16**	**0.02**	1.11	4.19	488
	55+	1.36	0.66	0.35	5.20	1.39	0.64	0.35	5.46	1.34	0.54	0.52	3.44	244
Migration status	native	ref.												3565
	emigrant	2.14	0.20	0.63	7.20	**6.78**	**0.02**	1.45	31.84	**3.06**	**0.01**	1.29	7.27	54
	immigrant	1.03	0.92	0.58	1.81	1.32	0.48	0.61	2.83	1.17	0.49	0.75	1.84	503
	visitor: born & live abroad	0.70	0.45	0.28	1.96	1.78	0.73	0.19	3.26	0.78	0.53	0.36	1.69	226
Any STI test in last 12 months	no	ref.												2249
	yes	1.15	0.62	0.67	1.95	**3.01**	0.00	1.56	5.84	**1.94**	**0.00**	1.42	2.66	2007
STI diagnoses	no diagnosis	ref.												3946
	1 diagnosis	0.71	0.36	0.34	1.48	**6.31**	**0.00**	2.10	18.05	1.24	0.43	0.72	2.14	310
	2 diagnoses	1.03	0.97	0.31	3.35	4.96	0.14	0.60	41.01	1.40	0.52	0.50	3.88	74
	3 diagnoses	1.07	0.95	0.14	8.10	1.00	–	–	–	1.44	0.73	0.19	10.88	18
	4 diagnoses	1.00	–	–	–	1.00	–	–	–	1.00	–	–	–	8
	5 diagnoses	6.04	0.12	0.62	58.66	1.00	–	–	–	8.15	0.07	0.84	78.82	4
	any STI diagnosis	0.82	0.52	0.46	1.49	**5.98**	**0.00**	2.23	16.06	1.31	0.26	0.82	2.09	414
Number of sex partners	no partner	ref.												269
	1 partner	0.64	0.38	0.24	1.72	4.82	0.13	0.62	37.34	1.37	0.46	0.59	3.17	819
	2–3 partners	0.78	0.59	0.31	1.96	3.73	0.21	0.47	29.34	1.34	0.48	0.59	3.07	981
	4–5 partners	0.79	0.63	0.30	2.08	4.57	0.16	0.56	37.45	1.49	0.36	0.64	3.51	626
	6–10 partners	0.67	0.43	0.25	1.80	**8.35**	**0.04**	1.08	64.42	1.62	0.26	0.70	3.73	701
	> 10 partners	0.99	0.89	0.40	2.47	**10.21**	**0.03**	1.30	79.90	**2.28**	**0.05**	1.01	5.14	748
Number of partners with condomless anal intercourse	no partner	ref.												1499
	1 partner	0.57	0.06	0.32	1.02	1.30	0.54	0.57	2.96	0.85	0.49	0.55	1.33	1108
	2–3 partners	1.13	0.65	0.67	1.90	**2.11**	**0.07**	0.95	4.68	1.36	0.16	0.89	2.07	839
	4–5 partners	0.67	0.46	0.24	1.92	**5.03**	**0.00**	1.92	13.16	1.50	0.22	0.79	2.85	235
	6–10 partners	1.17	0.75	0.45	3.05	2.94	0.10	0.80	10.67	1.79	0.09	0.92	3.50	182
	> 10 partners	**2.68**	**0.02**	1.14	6.30	**12.84**	**0.00**	3.75	43.93	**3.55**	**0.00**	1.83	6.88	106
Type of partners in last 6 months	steady partner(s)	ref.												653
	non-steady partner(s)	1.18	0.65	0.58	2.38	1.08	0.86	0.49	2.35	1.12	0.66	0.68	1.85	1297
	s + ns partner(s)	1.31	0.42	0.68	2.55	1.00	1.00	0.49	2.04	1.23	0.39	0.77	1.97	2001
	no partner	1.67	0.35	0.56	4.97	0.22	0.15	0.03	1.71	0.78	0.60	0.31	1.95	216
Anal sex in last 6 months	no anal sex	ref.												857
	anal intercourse (with condom)	0.87	0.65	0.47	1.59	1.21	0.67	0.50	2.95	1.12	0.65	0.69	1.84	1005
	condomless anal intercourse	0.80	0.43	0.47	1.38	**1.95**	**0.05**	0.99	3.85	1.30	0.21	0.86	1.98	2498
Type of partner at last anal sex	steady partner	ref.												1880
	non-steady partner	1.01	0.96	0.65	1.67	1.04	0.07	0.95	3.17	1.30	0.13	0.93	1.82	1760
	more than one partner	**2.06**	**0.04**	1.03	4.11	2.33	0.13	0.77	7.04	**2.22**	**0.01**	1.24	3.97	207
Sexual role with last AI partner	top	ref.												1324
	bottom	1.11	0.71	0.64	1.94	1.07	0.93	0.49	1.94	0.98	0.93	0.65	1.48	1265
	versatile	**2.08**	**0.00**	1.27	3.42	1.00	1.00	0.48	2.09	**1.61**	**0.02**	1.09	2.37	1035
Serostatus disclosure to last AI partner	no disclosure	ref.												2400
	disclosure	**0.27**	**0.00**	0.15	0.48	1.18	0.82	0.56	2.06	**0.50**	**0.00**	0.33	0.75	1262
Number of drugs consumed at last anal sex	0	ref.												1959
	1	**2.31**	**0.00**	1.37	3.91	0.91	0.75	0.50	1.66	**1.50**	**0.03**	1.04	2.16	1394
	2	**2.98**	**0.00**	1.58	5.61	0.99	0.99	0.41	2.42	**1.74**	**0.02**	1.08	2.79	516
	3	**4.30**	**0.00**	2.03	9.09	1.00	–	–	–	**2.31**	**0.01**	1.19	4.49	167
	4	**5.59**	**0.00**	1.79	11.80	2.04	0.50	0.26	15.97	**3.33**	**0.00**	1.46	7.55	76
	5	**4.69**	**0.02**	1.32	16.70	1.00	–	–	–	2.66	0.11	0.80	8.87	40
	6	**9.38**	**0.01**	1.89	54.64	7.66	0.07	0.83	70.60	**8.19**	**0.00**	2.25	29.82	15
	7	4.17	0.18	0.51	34.29	1.00	–	–	–	2.98	0.30	0.38	23.46	12
	8	1.00	–	–	–	1.00	–	–	–	1.00	–	–	–	6
	9	**18.76**	**0.02**	1.64	214.37	1.00	–	–	–	**10.93**	**0.04**	1.12	106.62	4
Type of drugs	no party drug	ref.												3875
	party	**2.90**	**0.00**	1.77	4.77	0.36	0.32	0.05	2.66	**2.04**	**0.00**	1.29	3.22	307
	no chemsex drug	ref.												4063
	chemsex	**2.14**	**0.04**	1.04	4.39	1.89	0.54	0.25	14.45	**2.42**	**0.01**	1.24	4.71	111
	no sexual performance substance	ref.												3326
	sexual performance substance	**2.41**	**0.00**	1.61	3.61	1.79	0.07	0.95	3.37	**2.15**	**0.00**	1.55	2.98	853
	no cannabis	ref.												3869
	cannabis	**2.20**	**0.01**	1.27	3.79	0.89	0.85	0.27	2.91	**1.75**	**0.02**	1.08	2.83	304
	no alcohol	ref.												2.324
	alcohol	**1.90**	**0.00**	1.26	2.84	0.70	0.23	0.40	1.25	1.33	0.07	0.98	1.80	1859
Satisfaction with sex life	unsatisfied	ref.												950
	satisfied	1.70	0.08	0.94	3.08	1.16	0.67	0.59	2.27	1.48	0.06	0.98	2.22	3.124

Compared with HIV uninfected survey participants, men assigned to the uHIVinc group were more likely to be 25–44 years of age (compared to the reference age group 18–24), and showed higher odds for the use of drugs during last anal sex, they were less likely to have disclosed their presumed negative HIV serostatus to their last anal sex partner(s), more likely to have been versatile during their last anal sex encounter, and more likely to have had more than 10 partners in the last 6 months with whom they had condomless anal intercourse.

Men assigned to the uHIVunk group were more likely to be older (age groups 35–44) than HIV-uninfected men who had not been tested for HIV in the last 12 months, to report any condomless anal intercourse in the last 6 months, and to have higher numbers of partners in the last 6 months with whom they had condomless anal sex, they were more likely to have been tested for and diagnosed with an STI in the last 12 months, and more likely to be an emigrant on home visit to his country of origin, but they were mostly inconspicuous in terms of substance use and most other potential explanatory variables.

In multivariate analysis assignment to the uHIVinc group remained significantly associated with age 25–34, and versatility, lack of serostatus disclosure, and use of party and sexual performance enhancing drugs during the last anal sex event (see Table 4). The only factors remaining associated with uHIVunk in multivariate analysis were age 35–54, higher number of partners with whom condomless anal sex had been practiced in the last 6 months, and more frequent STI testing in the last 12 months.

**Table 4:** Multivariate multilevel models to estimate associations between undiagnosed^a^ HIV infection and demographic and behavioural parameters among participants of the Sialon II study

		Odds Ratio	*p*-value	[95% Conf. Interval]	
uHIVinc (*n* = 1713)					
Age group	18–24	ref.			
	25–34	**2.27**	**0.04**	1.03	4.99
	35–44	1.62	0.29	0.67	3.90
	45–54	1.37	0.56	0.48	3.96
	55+	1.08	0.91	0.26	4.46
Sexual role with last AI partner	top	ref.			
	bottom	1.12	0.73	0.60	2.07
	versatile	**2.05**	**0.01**	1.18	3.55
Type of drugs during last anal sex	no use of ecstasy, cocaine, amphetamine	ref.			
	ecstasy, cocaine, amphetamine	**2.22**	**0.02**	1.17	4.21
	no use of sexual performance substances (poppers, erectile dysfunction medication)	ref.			
	sexual performance substances (poppers, erectile dysfunction medication)	**1.96**	**0.01**	1.17	3.28
Serostatus disclosure to last AI partner	disclosure	ref.			
	no disclosure	**3.94**	**0.00**	2.14	7.27
_cons		*0.01*	*0.00*	*0.00*	*0.01*
city		*0.40*	*–*	*0.11*	*1.45*
uHIVunk (*n* = 1639)					
Age group	18–24	ref.			
	25–34	2.00	0.16	0.76	5.27
	35–44	**3.73**	**0.01**	1.41	9.84
	45–54	**3.31**	**0.04**	1.08	10.12
	55+	0.90	0.93	0.10	7.84
Number of partners with condomless anal intercourse	no partner	ref.			
	1 partner	1.46	0.38	0.63	3.36
	2–3 partners	**2.66**	**0.02**	1.17	6.04
	4–5 partners	**6.08**	**0.00**	2.26	16.40
	6–10 partners	2.01	0.38	0.43	9.44
	> 10 partners	**12.83**	**0.00**	3.60	45.65
STI testing in last 12 months	no testing	ref.			
	any STI testing	**3.61**	**0.00**	1.74	7.50
_cons		*0.02*	*0.00*	*0.01*	*0.07*
city		*0.00*	*–*	*–*	*–*
uHIV (*n* = 3745)					
Age group	18–24	ref.			
	25–34	**2.36**	**0.00**	1.33	4.19
	35–44	**2.22**	**0.01**	1.19	4.13
	45–54	1.90	0.09	0.91	3.94
	55+	0.85	0.78	0.27	2.66
HIV test in last 12 months and knowing the result	not tested	ref.			
	tested and knowing the result	**1.51**	**0.03**	1.04	2.19
Number of partners with condomless anal intercourse	no partner	ref.			
	1 partner	0.85	0.50	0.54	1.35
	2–3 partners	1.28	0.30	0.81	2.03
	4–5 partners	1.35	0.39	0.68	2.69
	6–10 partners	1.56	0.25	0.74	3.29
	> 10 partners	**2.80**	**0.01**	1.34	5.85
Type of drugs during last anal sex	no use of sexual performance substances (poppers, erectile dysfunction medication)	ref.			
	sexual performance substances (poppers, erectile dysfunction medication)	**1.91**	**0.00**	1.32	2.76
_cons		*0.01*	*0.00*	*0.01*	*0.02*
city		*0.32*	*–*	*0.10*	*1.01*

^a^the three models estimate associations in three groups:

uHIVinc – undiagnosed HIV in a group of men reporting a last negative HIV test result within the previous 12 months

uHIVunk – undiagnosed HIV in a group of men who never tested for HIV or whose last negative HIV test result is older than 12 months

uHIV – undiagnosed HIV in the combined group of men irrespective of the time of the last negative HIV test

Education, migration status, outness, frequency of visiting gay sex venues in the last 6 months, partnership status, type of partner for the last anal intercourse, condom use during last anal intercourse, and sexual role during last anal intercourse were not significantly different between men with and without undiagnosed HIV infection.

## Discussion

Approximately one third of the study participants who were living with HIV and for whom their HIV status knowledge could be assessed were unaware of being infected. This is much higher than proportions reported from some modelling studies or estimates reported to ECDC for Dublin Declaration monitoring [2, 25, 26]. This apparent contradiction is likely explained by an age related effect in our sample: as we can show in our analyses, the proportion of undiagnosed HIV is highly age-dependent. A large proportion of MSM living with HIV in the Western European countries, where the HIV epidemic amongst MSM started already in the 1980s, is already older than 40 years. These higher age groups are underrepresented among the visitors of gay venues that often cater to younger MSM clients. When the different age composition of the Sialon sample and the MSM population in modelling studies are considered, the results in terms of the proportions of undiagnosed infections are essentially comparable [own unpublished comparisons between modelling results of the German undiagnosed fraction and Sialon results for Hamburg]. Contrastingly, in Eastern European countries, where the HIV epidemic among MSM is more recent and the fraction of older infections in aging survivors is much smaller, the Sialon results are comparable with modelling studies [27]. Another aspect that needs to be considered when comparing Sialon II results with national modelling studies is that Sialon II was conducted in large cities while modelling studies include whole countries. Regardless, our findings underline that in many settings where MSM congregate and seek sexual partners, a considerable proportion of those who are living with HIV are unaware of their HIV status.

Our analysis further shows that men with an undiagnosed HIV infection are a heterogeneous group of people. In our European multi-city sample, approximately two-third of those with undiagnosed HIV infection reported to have received a negative HIV test result in the previous 12 months, indicating the relatively recent acquisition of the infection and substantial incidence in this group. Moreover, this subgroup of men appears to test more frequently and be aware of risks. Taking this into account, the probability is high that many of them would have been tested again and diagnosed in the near future. It might also be that some of them tested in the HIV window period and received a false negative test result. To improve early HIV diagnosis in this group, men with these characteristics presenting for HIV testing should be offered laboratory testing with 4th generation HIV antigen/antibody tests to increase the probability to detect recent infections. If sufficient resources are available, even targeted PCR testing could be considered if this subgroup can be identified among the clients of the testing facilities, e.g. based on a combined symptoms and behaviours score [28, 29].

The men with undiagnosed infection following a negative test within the past 12 months had high odds of having used recreational drugs during their last anal sex encounter and high odds of not discussing their HIV status with the last anal sex partner(s) [30]. Because viral load and transmissibility of HIV are very high during the phase of acute HIV infection [31–33], many of their recent sexual partners may have been at high risk for acquiring HIV infection if they engaged in condomless anal intercourse relying on an assumed negative HIV status. In the literature, the associations between repeat testing and risk behaviours are complex. Receiving a negative result may trigger different reactions from reassurance in safe practices to feeling lucky or invulnerable, or reinforce risky behaviour that is associated with a subsequent higher frequency of unprotected sex [34].

These findings clearly point to the need of recommending more frequent testing in selected groups of MSM, especially to those using recreational drugs. More importantly, the testers could be considered as primary target group for HIV pre-exposure prophylaxis (PrEP) to avoid HIV infection in the first place, as also suggested by other authors [35].

Approximately one-third of the men with undiagnosed HIV in the Sialon II sample infrequently test for HIV, although they tend to have multiple condomless anal sex encounters. Higher proportions were observed particularly in the four RDS cities, which may suggest that more hidden subgroups within the MSM populations were reached (see also Limitations). This, from a public health perspective, is an advantage of this sampling methodology compared to TLS method and probably to National HIV surveillance systems as well. While the study was not designed to answer the research question on identifying characteristics and behaviours of undiagnosed HIV-infected participants, only number of partners with whom condomless anal intercourse was practiced and more frequent STI testing was associated with the outcome variable (undiagnosed HIV infection) in this group. While age was significantly and independently associated with being undiagnosed in this group, more research will be necessary to characterize MSM living with undiagnosed HIV infection who do not test frequently for this infection in order to develop evidence-based interventions to increase test uptake. However, in the bivariate analysis we also found high odds for having been diagnosed with a STI during the last 12 months in this group. This strongly suggests that contrary to guidelines and recommendations HIV testing had not been offered or not been conducted in the context of these STI diagnoses. We are unable to determine whether this missed opportunity for an earlier diagnosis of HIV is related to a lack of discussion and disclosure of sexual orientation with the STI test and treatment provider or to a lack of compliance with testing guidelines by the STI treatment providers.

Partnership status and type of partner for last anal intercourse were not significantly associated with undiagnosed HIV, suggesting that condomless sex within steady partnerships may not always be as safe as people tend to assume, particularly if HIV status has not been checked mutually and/or if condomless anal sex is practiced concurrently with non-steady partners.

### Limitations

For correct interpretation of our findings it must be considered that we report on associations with undiagnosed HIV infections in a very specific group. Factors associated with undiagnosed HIV may partly be different from factors associated with transmission risk, because a part of those who acquire HIV will be diagnosed and detected early. For MSM who infrequently test for HIV it may be difficult to detect behavioural correlates for their infection risk because we asked for behaviours in the previous 6 months. The moment when these men acquired HIV may be longer ago and their behaviour may have changed. MSM who have never been tested for HIV may be underrepresented in our sample. Never tested MSM are often less integrated into gay communities and rarely visit gay venues; this explains why they would have a lower chance to be recruited in our study, at least when considering the cities where a TLS approach has been adopted to enrol study participants [36]. This means that our uHIVunk group may mainly represent pattern 2 testing (triggered by health concerns not immediately related to acute HIV disease or transmission risk awareness) and less pattern 3 testing. A further limitation is that HIV status knowledge was based on self-reports and some participants may have felt uncomfortable reporting their HIV status in the questionnaire. Underreporting of a positive HIV status would have weakened any association we found between being undiagnosed and other factors.

## Conclusions

Our study findings reinforce the recommendations for healthcare provider-initiated HIV testing when certain indicator diseases such as STIs are diagnosed. The findings may also inform community-based low-threshold HIV testing strategies such as home-collection sampling and test promotion campaigns to reduce the proportion of the hidden HIV epidemic. Such strategies should include certain elements of information (e.g. on the sensitivity of different tests during acute HIV infection), focus on interpersonal skills and community norms (e.g. communication with sexual partners about serostatus) and highlight additional risks associated with recreational drug use, while recognising the diversity of MSM with undiagnosed HIV across Europe. In addition, novel strategies such as home-testing should be discussed in the light of safeguarding linkage to care [37]. Since data were collected in different European cities, the findings allow for a high degree of tailoring local prevention campaigns, i.e. developing targeted HIV and STI testing campaigns considering the local contexts in both community-based HIV testing and counselling and advice offered at such HIV testing sites [38, 39].

More importantly, tailored strategies based on the established HIV testing patterns should be embedded within an overall combined prevention approach [40], which should include the addition of PrEP to the available effective prevention tools [40–42] for instance for those MSM reporting condomless anal sex with multiple partners in the last 6 months.

## Additional files

Additional file 1: English language version of the self-administered questionnaire filled-in by the study participants. (PDF 235 kb)

Additional file 2: Sialon II dataset used for the analysis presented in this manuscript. (CSV 11362 kb)

Additional file 3: Stata do-file of the analysis. (TXT 10 kb)

## Abbreviations

AI: Anal intercourse
CD4: T-helper lymphocytes
CLIA: Chemoluminescence-Immunoassay
ECDC: European Centre for Disease Prevention and Control
ELISA: Enzyme Linked Immuno Sorbent Assay
EU: European Union
GHB: γ-hydroxybutric acid
HIV Ag/Ab: HIV Antigen/Antibody
HIV: Human Immunodeficiency Virus
IgG: Immunoglobulin G
MSM: Men having sex with men
nHIV: HIV-uninfected
OF: Oral fluid
pHIV: Previously diagnosed with HIV infection
PrEP: Pre-exposure prophylaxis
RDS: Respondent-driven-sampling
STI: Sexually transmitted infection
TLS: Time-location-sampling
uHIV: HIV-infected but as yet undiagnosed
uHIVinc: Incident HIV infection - negative test result reported within 12 months before the study specimen was collected
uHIVunk: HIV infection of unknown onset
UK: United Kingdom
WHO: World Health Organization
WHO-ERC: WHO Ethics Review Committee
WHO-RP2: WHO Research Project Review Panel
